# Supplementary material for: SCI-YOLO11: An improved defect detection algorithm for transmission line insulators based on YOLO11
Source: PLoS One. 2025 Oct 29;20(10):e0322561. doi: 10.1371/journal.pone.0322561 (PMC12571245; doi:10.1371/journal.pone.0322561)
Supplement: Attachment — Submitted filename: Response to Reviewers.docx [file pone.0322561.s002.docx]

Dear Editor and Associate Editor,

Thank you very much for taking the time to review and provide constructive feedback on my manuscript. I am sincerely grateful for the detailed and valuable suggestions you have given. Your insightful comments have greatly improved the quality of my work and provided me with a deeper understanding of the necessary improvements.

In response to your suggestions, I have made the following revisions to the manuscript:

Formatting and Style: I carefully reviewed the manuscript to address grammatical, punctuation, and formatting errors. I also ensured compliance with the PLOS ONE style requirements, including file naming, line spacing, and the removal of numbered titles.

Figures and Tables: I added captions for missing figures (e.g., Figure 5) and ensured all figures and tables are properly referenced in the text. The issues related to Figures 11-14 and Tables 1-2 have been resolved.

References: I updated the references by including recent works (2022–2024) and corrected missing or inaccurate citations, such as Reference [3].

Code Availability: I uploaded the source code to GitHub (https://github.com/Xiaobaisci/SCI-yolo11.git) and included this information in the abstract for transparency and reproducibility.

Algorithm Explanation: I expanded the explanation of the Wise-IoU-V3 loss function and SPD-Conv module, providing clearer derivations and detailed discussions.

Related Work Section: I revised the related work section to explicitly differentiate the proposed method from existing approaches, marking the changes for clarity.

In the **Revised Manuscript with Track Changes** file, the changes are marked as follows: red indicates the original content before modification, and green highlights the revised content.
In the **Response to Reviewers** file, the original comments by reviewers are in red, the content before modification is highlighted in yellow, and the revised content is highlighted in green.

I hope the revised manuscript addresses all concerns effectively. Please let me know if there are any additional revisions required. Once again, I sincerely appreciate your time and effort in reviewing my submission.

Best regards,

Wang Yuqian

sunbaby0830@sohu.com

Responeses to Editor (Original comments by reviews are in red color, the content before modification is highlighted in yellow, and the revised content is highlighted in green.)

1. **Comment:**

Please ensure that your manuscript meets PLOS ONE's style requirements, including those for file naming. The PLOS ONE style templates can be found at https://journals.plos.org/plosone/s/file?id=wjVg/PLOSOne_formatting_sample_main_body.pdf and https://journals.plos.org/plosone/s/file?id=ba62/PLOSOne_formatting_sample_title_authors_affiliations.pdf

1. **Reply:**

Thank you very much for your valuable feedback. I have carefully reviewed the PLOS ONE style requirements and made the necessary revisions to ensure compliance. Specifically, I optimized the formatting of the author information to align with the guidelines, corrected improper punctuation usage (e.g., adding spaces after punctuation marks to improve readability), adjusted the manuscript’s line spacing to double spacing as required, and removed numbering from section titles, as it is not included in the official template. To make the revisions clear and transparent, the changes in the manuscript have been highlighted, with the original text marked in yellow and the revised content shown in green. Additionally, I conducted a thorough review of the entire manuscript to address any spelling errors, punctuation mistakes, or other formatting inconsistencies. I sincerely hope these adjustments meet the journal’s requirements and greatly appreciate your guidance and support.

Junyan Wang^1^, Yuqian Wang^2*^,Xun Li^3^, Baoxi Yuan^4^,Miaomiao Wang^5^

^1^Xijing University,Xi’an,Shaanxi,710123,china

^2^Graduate Office,Xijing University,Xi'an,Shaanxi 710123,China

^3^Xijing University,Xi’an,Shaanxi,710123,china

^4^Xi’an Key Laboratory of High Precision Industrial Intelligent Vision Measurement Technology, Xijing University, Xi’an, Shaanxi, China,

^5^North China Electric Power University,Baoding,071051,china

*** Correspondence:**
Corresponding Author: Yuqian Wang
sunbaby0830@sohu.com

Junyan Wang^1^, Yuqian Wang^2*^,Xun Li^1^, Baoxi Yuan^3^,Miaomiao Wang^4^

1 Xijing University, Xi’an, Shaanxi,710123, china

2Graduate Office, Xijing University, Xi'an, Shaanxi 710123, China

3Xi’an Key Laboratory of High Precision Industrial Intelligent Vision Measurement Technology, Xijing University, Xi’an, Shaanxi, China,

4North China Electric Power University, Baoding, 071051, china

***** sunbaby0830@sohu.com

**2.Comment:** Please note that PLOS ONE has specific guidelines on code sharing for submissions in which author-generated code underpins the findings in the manuscript. In these cases, we expect all author-generated code to be made available without restrictions upon publication of the work. Please review our guidelines at https://journals.plos.org/plosone/s/materials-and-software-sharing#loc-sharing-code and ensure that your code is shared in a way that follows best practice and facilitates reproducibility and reuse.

**2.Reply:**

Thank you for bringing this to my attention. In response to your comment, I have ensured that the author-generated code is shared in compliance with PLOS ONE's guidelines for code sharing. The code has been uploaded to GitHub at the following link: https://github.com/Xiaobaisci/SCI-yolo11.git. This information has also been included in the manuscript’s abstract to facilitate reproducibility and reuse. I hope this meets the journal’s requirements, and I appreciate your guidance on this matter.

Our code has been uploaded to: https://github.com/Xiaobaisci/SCI-yolo11.git.

**3. Comment:** We suggest you thoroughly copyedit your manuscript for language usage, spelling, and grammar. If you do not know anyone who can help you do this, you may wish to consider employing a professional scientific editing service.

**3. Reply:**

Thank you for the suggestion regarding language usage, spelling, and grammar. I have carefully reviewed the manuscript to ensure it meets the required standards. For example, in the sentence "Complex Background Interference Issue: Images captured during insulator inspections frequently contain intricate backgrounds including wires trees along with weather variations that hinder effective recognition of targets," I revised "wires trees" to "wires, trees" to clearly express the list and avoid ambiguity. Additionally, I corrected instances where non-English quotation marks (e.g., “’ ”) were mistakenly used and replaced them with proper English punctuation. I hope these efforts have improved the clarity and accuracy of the manuscript.

4. **Comment:** For studies involving third-party data, we encourage authors to share any data specific to their analyses that they can legally distribute. PLOS recognizes, however, that authors may be using third-party data they do not have the rights to share. When third-party data cannot be publicly shared, authors must provide all information necessary for interested researchers to apply to gain access to the data. (https://journals.plos.org/plosone/s/data-availability#loc-acceptable-data-access-restrictions)

For any third-party data that the authors cannot legally distribute, they should include the following information in their Data Availability Statement upon submission:

1) A description of the data set and the third-party source

2) If applicable, verification of permission to use the data set

3) Confirmation of whether the authors received any special privileges in accessing the data that other researchers would not have

4) All necessary contact information others would need to apply to gain access to the data.

**4.Reply:**

Thank you for your comments regarding data availability. The dataset used in this study is not third-party data but rather our proprietary dataset. It has been uploaded to the Kaggle platform for researchers to access and use.

The dataset can be accessed at the following link: [https://www.kaggle.com/datasets/jmwangdeep/junyan-wang-insulator-dataset].

We hope that the availability of this dataset will contribute to research reproducibility and transparency. Please feel free to contact us if further clarification is needed.

This dataset has been made publicly available on Kaggle (https://www.kaggle.com/datasets/jmwangdeep/junyan-wang-insulator-dataset) to support further research in related fields.

5. **Comment:** PLOS requires an ORCID iD for the corresponding author in Editorial Manager on papers submitted after December 6th, 2016. Please ensure that you have an ORCID iD and that it is validated in Editorial Manager. To do this, go to ‘Update my Information’ (in the upper left-hand corner of the main menu), and click on the Fetch/Validate link next to the ORCID field. This will take you to the ORCID site and allow you to create a new iD or authenticate a pre-existing iD in Editorial Manager.

**5. Reply:**

Thank you for the reminder regarding the ORCID iD requirement for the corresponding author. I have ensured that my ORCID iD (0009-0008-8664-2720) is correctly entered and validated in Editorial Manager by following the provided instructions. Please let me know if any further action is needed.[0009-0008-8664-2720](javascript:popup_orcidDetail('https://orcid.org','0009-0008-8664-2720');)

6. **Comment:** Please ensure that you refer to Figures 11, 12, 13, 14 in your text as, if accepted, production will need this reference to link the reader to the figures.

6. **Reply:**

I sincerely apologize for the oversight regarding the references to Figures 11, 12, 13, and 14 in the text. I have now carefully reviewed the manuscript, ensured that these figures are properly captioned, and added the necessary references to them in the text. Additionally, I have standardized all figure references throughout the manuscript to maintain consistency and clarity. Thank you for bringing this to my attention, and I appreciate your understanding.

7. **Comment:** Please include a caption for figure 5.

**7. Reply:**

Thank you for the reminder. I have added an appropriate caption for Figure 5 in the manuscript. Please let me know if any further adjustments are needed.

**Fig5. The Process of Data Collection and Annotation by Drones**

8. **Comment:** We note you have included a table to which you do not refer in the text of your manuscript. Please ensure that you refer to Table 1, 2 in your text; if accepted, production will need this reference to link the reader to the Table.

**8. Reply:**

Thank you very much for pointing out this issue. I sincerely apologize for the oversight regarding the missing references to Table 1 and Table 2 in the text. I have carefully reviewed the manuscript, ensured that appropriate captions have been added to both tables, and included the necessary references to them in the text. I greatly appreciate your attention to detail and your kind reminder, and I hope the revisions now meet the journal’s requirements.

Responeses to reviews(Original comments by reviews are in red color, the content before modification is highlighted in yellow, and the revised content is highlighted in green.)

Reviewer#1:

**1.Comment:**Check for grammatical, punctuation, and spelling errors.

**1.Reply:**

Thank you for the valuable suggestion. I have carefully reviewed the manuscript for grammatical, punctuation, and spelling errors. Specifically, I corrected instances where non-English punctuation marks such as “’” were mistakenly used and replaced them with proper English punctuation. Additionally, I ensured that spaces are included after periods and other punctuation marks to improve readability. For example, in the sentence "Images captured during insulator inspections frequently contain intricate backgrounds including wires trees along with weather variations that hinder effective recognition of targets," I revised "wires trees" to "wires, trees" to clearly express the list and avoid ambiguity. I hope these revisions address the concerns and enhance the manuscript’s clarity and accuracy.

**2.Comment:**Use more significant 4 to 6 keywords.

**2.Reply:**

Thank you for the valuable feedback regarding the keywords. We have updated them to better reflect the focus of our work. The revised keywords are: Insulator Defect Detection, YOLO11 Improvement, Small Object Detection, Lightweight Design. We hope these provide a more precise representation of our study's contributions.

**3. Comment:** Use the references correctly. Some in-text citations are missing.

**3. Reply:**

Thank you for pointing out the issue with missing in-text citations. I sincerely apologize for the oversight. For example, in the sentence "Among these challenges lies the issue of detecting small targets like insulator defects; their characteristics may be lost within deep convolutional networks (CNNs), leading to missed detections or false positives [3]," the reference [3] was initially missing but has now been corrected. Additionally, I conducted a thorough review of all references in the manuscript, updated missing citations, and ensured their proper usage throughout the text. I hope these revisions address the concern, and I greatly appreciate your attention to this matter.

Among these challenges lies the issue of detecting small targets like insulator defects; their characteristics may be lost within deep convolutional networks (CNNs), leading to missed detections or false positives [3].

The rapid development of deep learning has provided new technological solutions to address these issues. For example, crime prediction research has successfully employed YOLOv8n for detecting small and intricate targets like weapons, achieving robust results even in complex scenarios [3].

[3] Liu, C., Wu, Y., Liu, J., & Han, J. (2021). MTI-YOLO: A light-weight and real-time deep neural network for insulator detection in complex aerial images. Energies, 14(5), 1426.

4. **Comment:** Add a summary table of previous works in related work section.

**4. Reply:**

Thank you for your constructive suggestion. Following your advice, we have added a summary table of previous works in the related work section to provide a clearer and more comprehensive comparison of methodologies, key improvements, and limitations in insulator defect detection research. This table helps highlight the contributions and innovations of our proposed SCI-YOLO11 algorithm. We hope this enhancement meets your expectations.

| Reference | Methodology & Key Features | Limitations |
| --- | --- | --- |
| [13] | YOLOv2+Classifier, UAV, surface condition analysis | Limited small object detection |
| [14] | AlexNet+Classifier, infrared imaging | Binary classification only |
| [15] | YOLOv3+FPN, improved speed & accuracy | High model complexity |
| [16]&[47] | YOLO+ECA-Net, lightweight, self-attention | Weak small object detection |
| [48]&[49] | YOLOv8+GhostNet, multi-scale fusion, multi-fault types | Limited defect diversity |
| [19]&[20] | Faster R-CNN, multi-scene robustness | High computational cost |
| OURS | lightweight design, SPDConv, SE, WIoU | Needs testing in diverse environments |

5. **Comment:** Figure 2 is not clear and readable.

**5. Reply:**

Thank you for your valuable feedback. We have redrawn Figure 2 using Visio to ensure better clarity and readability. We hope the updated version meets your expectations.

6. **Comment:** Improve figure 3 quality.

**6. Reply:**

Thank you for your valuable feedback. We have redrawn Figure 3 using Visio to ensure better clarity and readability. Additionally, we have included computational details in the "Attention Mechanism: SE Attention" section to enhance its informativeness. We hope the updated figure meets your expectations.

7. **Comment:** Use updated references. You can cite this paper

M. R. Islam et al., "Crime Prediction by Detecting Violent Objects and Activity Using Pre-Trained YOLOv8n and MoViNet A0 Models," 2023 International Conference on Modeling & E-Information Research, Artificial Learning and Digital Applications (ICMERALDA), Karawang, Indonesia, 2023, pp. 44-49, doi: 10.1109/ICMERALDA60125.2023.10458156.

**7. Reply:**

Thank you for your valuable suggestion. I have updated the references in my manuscript to ensure accuracy and relevance.

Additionally, I have incorporated the recommended citation:

M. R. Islam et al., "Crime Prediction by Detecting Violent Objects and Activity Using Pre-Trained YOLOv8n and MoViNet A0 Models," 2023 International Conference on Modeling & E-Information Research, Artificial Learning and Digital Applications (ICMERALDA), Karawang, Indonesia, 2023, pp. 44-49, doi: 10.1109/ICMERALDA60125.2023.10458156.

The findings in this paper demonstrate the adaptability and effectiveness of deep learning models in small object detection, offering valuable insights for insulator defect recognition.

For example, crime prediction research has successfully employed YOLOv8n for detecting small and intricate targets like weapons, achieving robust results even in complex scenarios [3]. These findings demonstrate the adaptability and effectiveness of deep learning models for small-object detection, providing valuable insights for insulator defect identification.

Reviewer#2:

**1.Comment:** What are the researcher's contributions? Please explain them in brief points.

**1.Reply:**

Thank you for your valuable feedback!

The main contributions of this study are as follows:

Dataset Construction: Constructed and publicly released a diverse insulator defect detection dataset to address the limitations of existing datasets and support further research in the field;

Model Architecture Optimization: Introduced the SPDConv module to optimize feature extraction, significantly improving small target detection accuracy while reducing model parameters;

Attention Mechanism Enhancement: Integrated the SE attention mechanism to enhance feature representation, significantly improving detection performance in complex backgrounds;

Loss Function Improvement: Introduced the WIoU loss function to optimize the bounding box regression process, enhancing the model's adaptability to low-quality samples and overall robustness.

**2.Comment:** The working steps of the proposed algorithm are not explained algorithmically.

**2.Reply:**

Thank you for your valuable suggestion. Below is an explanation and improvement of the working steps of the proposed algorithm:

2.1 The SPD-Conv (Spatial-to-Depth Convolution) module is designed to enhance the feature extraction capabilities of low-resolution images and small targets by integrating spatial information into the channel dimension. The specific implementation process is shown in Table 2.

(1) Space-to-Depth Transformation:

Given an input feature map X with dimensions S×S×C_1_, the Space-to-Depth (SPD) operation divides the feature map into ${scale}^{2}$ sub-regions f_i,j_, where each sub-region is defined as show in Eq 1:

$f_{i,j}=X[i:S:sacle,j:S:scale,:]$	(1)

Here,i,j∈{0,1,...,scale-1}. Each sub-region has dimensions S/scale×S/scale×C_1_. These sub-regions are concatenated along the channel dimension to generate a new feature map X’, with dimensions as show in Eq 2:

$X^{'}(\frac{S}{scale},\frac{S}{scale},C_{1}\times{scale}^{2})$	(2)

This transformation reduces the spatial resolution while increasing the channel depth, effectively preserving rich spatial information.

(2) Non-strided Convolution:

The transformed feature map X’ is subsequently passed through a non-strided convolutional layer to extract enhanced features. The convolution operation is defined as show in Eq 3:

$Y=X^{'}*W+b$	(3)

Where W and b represent the convolutional kernel weights and bias terms, respectively. This process captures high-level semantic information and further optimizes feature representation. The final output feature map Y has dimensions: Y(s/scale,s/scale,C_2_), Here, C_2_ represents the number of convolutional kernels. In this study, the SPD-Conv module replaces the standard convolution in YOLO, enhancing the model's performance in detecting small objects and low-resolution targets. By incorporating the space-to-depth mechanism, the module captures finer-grained details, thereby improving the model's ability to detect small targets in complex backgrounds.

Table 2. Algorithm operation steps

| Step | Description |
| --- | --- |
| input | Feature map $X$ of size ($S\times S\times C_{1}$)  Downsampling factor $Scale$  Convolution kernel $W$  Output channel size $C_{2}$ |
| 1 | Split feature map $X$ into ${Scale}^{2}$ sub-feature maps:  $f_{i,j}=X[i:S:sacle,j:S:scale,:]$ |
| 2 | Concatenate all sub-feature maps along the channel dimension:  $X^{'}=Concatenate\left( f_{0,0},,f_{0,1}{\ldots,f}_{scale-1,scale-1} \right)$  The concatenated $X^{'}$ has size $(\frac{S}{scale},\frac{S}{scale},C_{1}\times{scale}^{2})$ |
| 3 | Apply non-strided convolution to $X^{'}$:  $Y=Conv(X^{'},W)$  The output feature map $Y$ has size ($\frac{S}{scale},\frac{S}{scale},C_{2}$) |
| output | feature map $Y$ of size ($\frac{S}{scale},\frac{S}{scale},C_{2}$) |

**2.2** In order to enhance the network's ability to capture features of small targets and critical regions, this paper introduces the Squeeze-and-Excitation (SE) attention mechanism between the SPPF layer and C2PSA layer in the backbone of SCI-YOLO11. Global average pooling is performed on the input feature map $F_{in}\in{\mathbb{\mathbb{R}}}^{C\times H\times W}$ to obtain a global feature vector along the channel dimension as shown in Eq 4:

$G=GAP\left( F_{in} \right)\Rightarrow G_{c}=\frac{1}{H\cdot W}\sum_{i=1}^{H} \sum_{j=1}^{W} F_{in}\left( c,i,j \right),c\in[1,C]$	(4)

The operation extracts global semantic information from each channel by compressing the spatial dimensions. Subsequently, the global feature G is input into a lightweight network for nonlinear transformation: $Z=\sigma(Relu(W_{2}\cdot tanh(W_{1}\cdot G)))$, Here,$W_{1}\in{\mathbb{\mathbb{R}}}^{\left( \frac{C}{16} \right)\times C}、W_{2}\in{\mathbb{\mathbb{R}}}^{\left( \frac{C}{16} \right)\times C}$ are learnable parameter matrices, $\tanh\left( \cdot\right)$ and $\sigma\left( \cdot\right)$ represent the tanh and sigmoid activation functions, respectively. The final output is to generate a weighted feature map by performing element-by-element multiplication, as shown in Eq 5

$F_{out}=F_{in}\cdot Z$	(5)

Fig 3**. Structure Diagram of the SE Attention Mechanism.**

## Loss functions

Despite existing work assuming that the training data consists of high-quality samples and focusing on enhancing the fitting capability of boundary box regression loss, blind optimization of low-quality samples may compromise localization accuracy. Although Focal-EIoU v1 was proposed to address this issue, its static focusing mechanism (FM) fails to fully leverage the potential of a dynamic non-monotonic FM—this mechanism can adjust gradient allocation based on training dynamics [28]. Based on this observation, we introduce a distance attention mechanism built upon the original Wise-IoU framework, proposing Wise-IoU-V3. (A schematic representation of this regression process is illustrated in Figure 4). This approach aims to achieve precise gradient prioritization for high-quality anchor boxes while mitigating the influence of low-quality samples.

The design objective is as follows: by dynamically adjusting gradient weights, we prioritize optimizing high-quality anchor boxes and suppressing the impact of low-quality samples.

(1) Design of Dynamic Non-Monotonic Focusing Mechanism

Traditional loss functions (such as Focal-EIoU) employ a static non-monotonic focusing strategy that cannot adapt to dynamic changes during training phases. To address this limitation, we introduce the degree of outlier (β), as shown in Eq 7, which quantifies the deviation between the current anchor box loss and the global average loss:

$\beta={L_{IOU}^{*}}/{\overline{L}_{IOU}}$	(7)

The term “L^*^_IOU_” refers to the IoU loss of the current anchor box, while "$\overline{L}_{IOU}$" denotes the global average loss, which is updated through exponential moving averages. Based on “β” we define a dynamic focusing coefficient as follows: r=β/(α^β-α^). when β=δ, r=1 represents the optimal gradient allocation point (maximum gradient gain).

Non-monotonicity:

When β>δ (low-quality samples), r approaches 0, suppressing the gradient,when β<δ (high-quality samples), γ increases linearly with $\beta$, prioritizing optimization.

The hyperparameters α and δ control the sensitivity and dynamic threshold of anomaly degree. Parameter δ is dynamically updated using momentum m as shown in Eq 8:

$\delta_{t+1}=m\cdot\delta_{t}+(1-m)\cdot\bar{L_{IOU}}$	(8)

At the initial stage of training, $\bar{L_{IOU}}\approx1$and m=0.1, which allows the model to focus on high-confidence regions for rapid convergence. In the later stages, as m increases, $\delta$ gradually stabilizes to the overall average value, thereby balancing ordinary samples and low-quality samples.

(2) Introduction of Distance-Aware Attention Weighting:

To enhance sensitivity to local regions with significant errors, a distance penalty term R_WIOU_ is defined, as shown in Eq 9

$R_{WIOU}=\exp\left[ \frac{\left( x-x_{gt} \right)^{2}+\left( y-y_{gt} \right)^{2}}{\left( W_{g}^{2}+H_{g}^{2} \right)^{*}} \right]$	(9)

Here, (W_g_,H_g_) represent the width and height of the bounding box for the ground truth, while ∗ denotes the detached calculation to avoid gradient instability. This weighting mechanism penalizes predictions that deviate significantly from the ground truth, encouraging the model to prioritize optimization in regions close to the target. The final loss function formula is shown in Eq 10:

$L_{Wise-IOUv3}=r\cdot L_{WiouV1}, L_{IOUv1}=R_{WIOU}\cdot L_{IOU}$	(10)

[28]Zhang, Y. F., Ren, W., Zhang, Z., Jia, Z., Wang, L., & Tan, T. (2022). Focal and efficient IOU loss for accurate bounding box regression. Neurocomputing, 506, 146-157.

**3. Comment:** Include a link to the dataset used in the reference list.

**3. Reply:**

Thank you for your thorough review and valuable suggestion! We completely agree with your recommendation to include the dataset link explicitly in the reference section. While we previously provided the dataset link in the Related Work section of the manuscript, your suggestion to add it to the references is more formal and enhances the scientific rigor and reproducibility of the work. In the revised manuscript, we have followed your advice and added the dataset details to the reference section, including its title, publication year, hosting platform, and direct link. The updated format is as follows:

The collection equipment consists of the DJI Matrice 300 RTK drone and the Zenmuse Z30 gimbal camera[29]

[29] Wang J. 2024. Junyan Wang Insulator Dataset. Kaggle. Available from: <https://www.kaggle.com/datasets/jmwangdeep/junyan-wang-insulator-dataset>

4. **Comment:** Clarify future work well for this work and all aspects

**4. Reply:**

Thank you for your valuable feedback! In future work, we plan to explore further possibilities for model lightweighting and precision improvement through advanced optimization methods, including neural architecture refinement and adaptive inference mechanisms, to enhance detection performance. Additionally, we aim to investigate the integration of environmental simulation effects, such as foggy weather conditions, to evaluate and improve the robustness of the model in adverse scenarios. These efforts will lay a solid foundation for advancing insulator defect detection methods and support the safe and stable operation of power transmission systems.

5. **Comment:** Include additional references to research published between 2022-2024

5.Reply: Thank you for your suggestion. I have added references to research published between 2022 and 2024 to ensure the manuscript reflects the latest advancements in the field. Examples include:

[3]M. R. Islam et al., "Crime Prediction by Detecting Violent Objects and Activity Using Pre-Trained YOLOv8n and MoViNet A0 Models," 2023 International Conference on Modeling & E-Information Research, Artificial Learning and Digital Applications (ICMERALDA), Karawang, Indonesia, 2023, pp. 44-49, doi: 10.1109/ICMERALDA60125.2023.10458156.

[14]Han G J, He M, Gao M G, et al. Insulator breakage detection based on improvedYOLOv5[J].Sustainability,2022,14(10):6066-6082.

[18]Zhang J R, Wei X, Zhang L X, et al. YOLO v7-ECA-PConv-NWD detects defectiveinsulators on transmission lines[J.Electronics, 2023,12(18):3969-3983.

[28]Zhang, Y. F., Ren, W., Zhang, Z., Jia, Z., Wang, L., & Tan, T. (2022). Focal and efficient IOU loss for accurate bounding box regression. Neurocomputing, 506, 146-157.

[25]Wei, L., Jin, J., Deng, K., & Liu, H. (2024). Insulator defect detection in transmission line based on an improved lightweight YOLOv5s algorithm. Electric Power Systems Research, 233, 110464.

Reviewer#3:

**1.Comment:** Correct all formatting issues, including "Error! Reference source not found.

**1.Reply:**

Thank you for bringing this issue to my attention. In the original manuscript, Reference [3] displayed the error "Error! Reference source not found." I sincerely apologize for this oversight. I have now updated Reference [3] and carefully reviewed the entire manuscript to ensure that all references are accurate, properly formatted, and free of errors.

**2.Comment:** Expand the explanation of the Wise-IoU-V3 loss function with a clearer derivation.

**2.Reply:**

Thank you for your suggestion. I have expanded the explanation of the Wise-IoU-V3 loss function in the manuscript, providing a clearer derivation and detailed discussion. This addition highlights the mathematical basis and design considerations of the loss function to ensure readers better understand its role in optimizing bounding box regression and addressing annotation quality issues.

Despite existing work assuming that the training data consists of high-quality samples and focusing on enhancing the fitting capability of boundary box regression loss, blind optimization of low-quality samples may compromise localization accuracy. Although Focal-EIoU v1 was proposed to address this issue, its static focusing mechanism (FM) fails to fully leverage the potential of a dynamic non-monotonic FM—this mechanism can adjust gradient allocation based on training dynamics [28]. Based on this observation, we introduce a distance attention mechanism built upon the original Wise-IoU framework, proposing Wise-IoU-V3. (A schematic representation of this regression process is illustrated in Figure 4). This approach aims to achieve precise gradient prioritization for high-quality anchor boxes while mitigating the influence of low-quality samples.

The design objective is as follows: by dynamically adjusting gradient weights, we prioritize optimizing high-quality anchor boxes and suppressing the impact of low-quality samples.

(1) Design of Dynamic Non-Monotonic Focusing Mechanism

Traditional loss functions (such as Focal-EIoU) employ a static non-monotonic focusing strategy that cannot adapt to dynamic changes during training phases. To address this limitation, we introduce the degree of outlier (β), as shown in Eq 7, which quantifies the deviation between the current anchor box loss and the global average loss:

$\beta={L_{IOU}^{*}}/{\overline{L}_{IOU}}$	(7)

The term “L^*^_IOU_” refers to the IoU loss of the current anchor box, while "$\overline{L}_{IOU}$" denotes the global average loss, which is updated through exponential moving averages. Based on “β” we define a dynamic focusing coefficient as follows: r=β/(α^β-α^). when β=δ, r=1 represents the optimal gradient allocation point (maximum gradient gain).

Non-monotonicity:

When β>δ (low-quality samples), r approaches 0, suppressing the gradient,when β<δ (high-quality samples), γ increases linearly with $\beta$, prioritizing optimization.

The hyperparameters α and δ control the sensitivity and dynamic threshold of anomaly degree. Parameter δ is dynamically updated using momentum m as shown in Eq 8:

$\delta_{t+1}=m\cdot\delta_{t}+(1-m)\cdot\bar{L_{IOU}}$	(8)

At the initial stage of training, $\bar{L_{IOU}}\approx1$and m=0.1, which allows the model to focus on high-confidence regions for rapid convergence. In the later stages, as m increases, $\delta$ gradually stabilizes to the overall average value, thereby balancing ordinary samples and low-quality samples.

(3) Introduction of Distance-Aware Attention Weighting:

To enhance sensitivity to local regions with significant errors, a distance penalty term R_WIOU_ is defined, as shown in Eq 9

$R_{WIOU}=\exp\left[ \frac{\left( x-x_{gt} \right)^{2}+\left( y-y_{gt} \right)^{2}}{\left( W_{g}^{2}+H_{g}^{2} \right)^{*}} \right]$	(9)

Here, (W_g_,H_g_) represent the width and height of the bounding box for the ground truth, while ∗ denotes the detached calculation to avoid gradient instability. This weighting mechanism penalizes predictions that deviate significantly from the ground truth, encouraging the model to prioritize optimization in regions close to the target. The final loss function formula is shown in Eq 10:

$L_{Wise-IOUv3}=r\cdot L_{WiouV1}, L_{IOUv1}=R_{WIOU}\cdot L_{IOU}$	(10)

[28]Zhang, Y. F., Ren, W., Zhang, Z., Jia, Z., Wang, L., & Tan, T. (2022). Focal and efficient IOU loss for accurate bounding box regression. Neurocomputing, 506, 146-157.

**3. Comment:** Improve the related work section by explicitly differentiating the proposed method from existing approaches.

**3. Reply:**

Thank you for your suggestion. I have updated the related work section to explicitly differentiate the proposed method from existing approaches. In the revised version, the distinctions are highlighted with clear comparisons in terms of feature extraction, attention mechanisms, and loss function design. Additionally, the changes are marked for clarity: yellow indicates the original content, and green represents the updated content.

Despite substantial advancements made thus far within existing research focused on insulating fault identification techniques—challenges such as difficulties associated with small target detections remain prevalent due largely due interference caused by intricate backdrops along with insufficient algorithmic lightweighting issues still persist—therefore further exploration into improved deep-learning-based methodologies holds considerable significance regarding enhancing capabilities surrounding these inspections specifically tailored towards identifying faults found among electrical components like those seen here involving insulated materials themselves .Drawing upon insights gained from prior researchers' experiences alongside established methodologies presented herein , this paper proposes an enhanced single-stage object-detection algorithm named SCI-YOLO11[21], explicitly optimized for tackling challenges related directly toward identifying flaws inherent amongst insulation systems Compared against conventional approaches, SCI-YOLO11 demonstrates clear advantages concerning precision, processing speeds & resource consumption levels alike . Due to the subpar quality of existing publicly available datasets and the limited variety of defect types, this paper presents a comprehensive dataset specifically designed for insulator defect detection. We captured images using DJI drones and enlisted experts in the field of electrical engineering for annotation. This self-constructed dataset ensures diversity and r-epresentativeness, and it has been made publicly accessible on the Kaggle platform:(https://www.kaggle.com/datasets/jmwangdeep/junyan-wang-insulator-dataset) thereby supporting ongoing scholarly inquiries into this domain .

The innovations embodied within SCI-YOLO11 can be summarized as follows:

(1) Model Structure Optimization: The introduction SPDConv[22]-based convolutional modules enhances capacity capturing characteristics pertaining smaller targets whilst simultaneously minimizing computational demands rendering deployment feasible even onto edge devices.

(2) Enhanced Attention Mechanism: Integration SE mechanism [23]within backbone networks bolsters expressive power leading ultimately improving overall accuracies when faced against challenging environments .

(3) Improved Loss Function : Design WIoU [24 ]loss function effectively balances training outcomes derived from high-quality versus low-quality samples alleviating discrepancies arising out imbalanced datasets consequently boosting models’ resilience/practicality substantially .

Through these enhancements implemented therein ,SCI-YOLO11achieves not only heightened levels fidelity but also maintains light-weightedness enabling real-time operational capacities further propelling advancements witnessed throughout industry practices revolving around inspecting electric infrastructures particularly focusing attention directed towards assessing integrity status exhibited among insulating materials utilized therein .

The innovations of SCI-YOLO11 are primarily reflected in the following aspects:

(1) The quality of existing publicly available datasets is generally low, and the types of defects are relatively limited. For instance, the CPLID dataset[23] only includes images of insulators under clear weather conditions and features a narrow range of defect types, which fails to comprehensively reflect the characteristics of insulators in power line samples. Therefore, this paper contributes a high-quality dataset specifically designed for insulator defect detection. We captured images using DJI drones and enlisted experts from the electrical field for annotation, ensuring diversity and representativeness within the dataset. This dataset has been made publicly available on Kaggle (https://www.kaggle.com/datasets/jmwangdeep/junyan-wang-insulator-dataset) to support further research in related fields.

(2) In terms of model structure optimization, we have incorporated the SPDConv [24] convolution module into the basic framework. This module utilizes deformable convolution kernels to dynamically adapt to the irregular shapes of insulator defects (such as flashover or loss), effectively enhancing the model's ability to capture features of small targets. Furthermore, compared to traditional convolution modules (such as standard convolutions and conventional deformable convolutions), ordinary convolutions struggle with precise detection of small target defects due to their limited size on transmission lines. The SPDConv significantly reduces the number of model parameters while maintaining accuracy, making it more suitable for deployment on edge devices. However, the method proposed in this paper achieves a favorable balance between feature extraction and model complexity; it not only improves detection accuracy but also substantially decreases hardware resource consumption.
(3) Enhancement of Attention Mechanism: We integrated the SE attention mechanism into the backbone network, which adaptively adjusts the feature channel weights to effectively enhance feature representation capabilities. This integration significantly improves model detection accuracy, particularly in complex background scenarios. Compared to previous methods, such as Wei et al. [25] who employed CBAM, our approach more effectively mitigates interference from complex backgrounds while maintaining a constant model parameter count and improving small object detection precision. Furthermore, by incorporating the SE mechanism [26] into the backbone network compared to baseline models without an attention mechanism, this study enhances feature representation ability and boosts detection accuracy in challenging backgrounds.

(4)Improvement of Loss Function: We introduced the WIoU [27] loss function to optimize the bounding box regression process, effectively balancing training outcomes between high-quality and low-quality samples. This adjustment alleviates detection errors caused by uneven sample quality. The improvement notably enhances both robustness and practicality of the model. In contrast to the CIOU loss function used in baseline models, WIoU not only focuses on bounding box fitting accuracy but also emphasizes the significant contribution of low-quality samples to training results, thereby reducing performance degradation due to data annotation biases. Additionally, WIoU further accelerates convergence speed while enhancing model adaptability in complex scenes.

In summary, through these various improvements, we propose a novel SCI-YOLO11 that achieves lightweight design and real-time performance while maintaining high precision levels. This advancement contributes positively to defect detection technology for insulators during practical power inspections. The summary table of previous work is presented in Table 1.

Table 1. Summary of Previous Work

| Reference | Methodology & Key Features | Limitations |
| --- | --- | --- |
| [11] | YOLOv2+Classifier, UAV, surface condition analysis | Limited small object detection |
| [12] | AlexNet+Classifier, infrared imaging | Binary classification only |
| [13] | YOLOv3+FPN, improved speed & accuracy | High model complexity |
| [14]&[17] | YOLO+ECA-Net, lightweight, self-attention | Weak small object detection |
| [18]&[19] | YOLOv8+GhostNet, multi-scale fusion, multi-fault types | Limited defect diversity |
| [20]&[21] | Faster R-CNN, multi-scene robustness | High computational cost |
| OURS | lightweight design, SPDConv, SE, WIoU | Needs testing in diverse environments |

[14]Han G J, He M, Gao M G, et al. Insulator breakage detection based on improvedYOLOv5[J].Sustainability,2022,14(10):6066-6082.

[18]Zhang J R, Wei X, Zhang L X, et al. YOLO v7-ECA-PConv-NWD detects defectiveinsulators on transmission lines[J.Electronics, 2023,12(18):3969-3983.

[19]He M, Qin L, Deng X, et al. MFI-YOLO: Multi-Fault insulator detection based on animproved YOLOv8[J. lEEE Transactions on Power Delivery, 2024, 39(1): 168-179

[23] Zhang ZD, Zhang B, Lan ZC, Liu HC, Li DY, Pei L, Yu WX. FINet: An insulator dataset and detection benchmark based on synthetic fog and improved YOLOv5. IEEE Transactions on Instrumentation and Measurement. 2022 Sep 27;71:1-8.
[24] Wei, L., Jin, J., Deng, K., & Liu, H. (2024). Insulator defect detection in transmission line based on an improved lightweight YOLOv5s algorithm. Electric Power Systems Research, 233, 110464.

4. **Comment:** Provide more details on the dataset’s diversity and representativeness.

**4. Reply:**

Thank you very much for pointing out the need to provide more details on the dataset’s diversity and representativeness. We sincerely apologize for this oversight and provide the following clarification: The dataset includes five typical types of insulator defects (Glass_Loss, Broken_disc, Glass_Dirty, Pollution_flashover, Polymer_dirty), with three representative subclasses selected for each type. Compared to existing datasets, which often focus on a single defect or coarse classification, our multi-dimensional balancing design significantly enhances generalization performance. Additionally, the dataset covers mainstream materials such as porcelain and glass, resulting in a total of 2,828 high-quality images. To ensure rigorous and reliable annotations, all samples were jointly labeled by three senior power system experts, achieving a high Kappa coefficient of 0.96. This systematic construction effectively addresses the limitations of existing datasets in terms of fine-grained defect categorization and material diversity. Thank you again for your valuable suggestion, which has helped us present the strengths and practicality of our dataset more comprehensively.

Fig 6**. Dataset for Insulator Defect Detection.**
